# *TLR7* and *TLR8* Gene Variations and Susceptibility to Hepatitis C Virus Infection

**DOI:** 10.1371/journal.pone.0026235

**Published:** 2011-10-13

**Authors:** Chiou-Huey Wang, Hock-Liew Eng, Kuei-Hsiang Lin, Cheng-Hsien Chang, Chi-An Hsieh, Yen-Li Lin, Tsun-Mei Lin

**Affiliations:** 1 Graduate Institute of Medicine, Kaohsiung Medical University, Kaohsiung, Taiwan; 2 Department of Clinical Laboratory, School of Medicine, College of Medicine, Kaohsiung Medical University, Kaohsiung, Taiwan; 3 Department of Ophthalmology, Kaohsiung Medical University, Kaohsiung, Taiwan; 4 Department of Medical Laboratory, E-DA Hospital/I-SHOU University, Kaohsiung, Taiwan; 5 Department of Medical Research, E-DA Hospital/I-SHOU University, Kaohsiung, Taiwan; 6 Department of Pathology, Chang Gung Memorial Hospital-Kaohsiung Medical Center, Chang Gung University College of Medicine, Kaohsiung, Taiwan; 7 Department of Medical Laboratory Science and Biotechnology, National Cheng Kung University, Tainan, Taiwan; Centers for Disease Control and Prevention, United States of America

## Abstract

Toll-like receptors (TLRs) play pivotal roles in the innate immune system and control inflammatory responses and adaptive immunity. We previously evaluated associations between *TLR7* and *TLR8* gene SNPs and susceptibility to hepatitis C virus (HCV) infection. Our results suggested that *TLR7IVS2-151G* and *TLR8-129G* alleles were present at higher frequency in males of an HCV-infected group as compared to a control group (24.1% vs. 14.4%, p = 0.028; 17.6% vs. 6.8%, p = 0.004, respectively). Based upon their recognition of single stranded viral RNA, this suggested that TLR7 and TLR8 played a significant role in anti-HCV immune responses. Here, we studied the functional effects of these polymorphisms by analyzing the mRNA expressions of *TLR7* and *TLR8* and cytokine production induced *ex vivo* by TLR7- and TLR8-specific agonists using whole blood of subjects with different genotypes. The percentage of CD14+ cells from those with an AG haplotype that expressed TLR7 and TLR8 was significantly lower, but higher in intensity compared to cells from those with GG and AC haplotypes. Cells from those with an AG haplotype produced more IFN-α and less amounts of pro-inflammatory cytokines upon stimulation. This suggests that variations in *TLR7* and *TLR8* genes might impair immune responses during HCV infection.

## Introduction

Toll-like receptors (TLRs) are important pathogen recognition receptors (PRRs) at the interface between a host and the environment and are key molecules for both innate and adaptive immunity [1]. TLR7 and TLR8, which share a high degree of structural similarity, are located in the membranes of the endosomal compartment and recognize viral single-stranded RNA (ssRNA) [2], [3], [4]. TLR genetic variants and downstream signalling molecules can influence the ability of affected individuals to respond adequately to TLR ligands, which can result in their altered susceptibility to or the course of infectious disease [5], [6], [7]. The number of reported genotypic profiles of TLRs is rapidly expanding. However, the number of identified functional TLR polymorphisms remains limited, and parts of some studies have divergent results regarding the significance of polymorphisms and even have contradictory observations [8].

We previously identified a single nucleotide polymorphism (SNP) at IVS2-151 of *TLR7* and a SNP resulting in a Met/Val change at a start codon *TLR8* associated (G/C) SNP at position −129 in the *TLR8* promoter region [9]. The polymorphism, *TLR7 IVS2-151 G>A*, changes the −151 nucleotide of the second intervening sequence (IVS-2 position −151) from G to A and it's function is still unclear. In our Chinese population, *TLR7* allele frequencies were A at 77%, and G at 22%, and *TLR8* allele frequencies were C at 16%, and G at 83%. However, to date there have been few studies on the functional effects of *TLR7* and *TLR8* gene polymorphisms.

Hepatitis C virus (HCV), a single-stranded RNA virus, infects more than 170 million of the world's population [10]. The clinical outcome of HCV infection is highly variable, and genetic factors involving innate immunity are likely to affect disease susceptibility and disease progression after infection. A key aspect of the antiviral innate immune response is the synthesis and secretion of type I interferons (IFN), such as IFN-α and IFN-β [11]. Based on their recognition of single stranded viral RNA, TLR7 and TLR8, both of which genes are located on the X chromosome [12], have been suggested to play important roles in antiviral immune responses induced by IFN and inflammatory cytokines.

Recent reports have indicated that robust TLR7 and TLR8 agonists decrease the amounts of HCV RNA in HCV-infected patients [13], [14]. The specific aims of this study were to evaluate possible associations between the susceptibility to HCV infection and *TLR7* and *TLR8* polymorphisms and to compare the expressions and functions of *TLR7* and *TLR8* variants.

## Results

### Allele and haplotype frequencies in chronic hepatitis C patients

The genotypes involving *TLR7* and *TLR8* SNPs of 264 patients with chronic HCV infection and 243 control subjects were analyzed. The distributions of the allele and haplotype frequencies for the *TLR7 IVS2-151 G>A* (rs179009) and *TLR8 -129G>C* (rs3764879) polymorphisms are summarized in Table 1. The frequency of *TLR7 IVS2-151G* was significantly higher in male chronic HCV infection patients than control subjects (24.1% versus 14.4%; p = 0.028), with an odds ratios (OR) of 1.89 (95% CI = 1.06 to 3.33). *TLR8 -129C* also had a significantly higher frequency in male chronic HCV infection patients than controls (17.6% versus 6.8%; p = 0.004); OR = 2.91 (95% CI = 1.38 to 6.13). However, no associations were found between chronic HCV infection and *TLR7* and *TLR8* polymorphisms among females (Table 1). Due to X skewing might affect the interpretation of data in female, we disregard TLR7 SNP heterozygous females from analysis [15] and got a significant difference in homozygous *TLR7 IVS2-151*genotypes among controls and hepatitis C female patients (p = 0.021; OR = 8.55,95% CI = 0.99 to 73.4).

**Table 1:** Genotype frequencies of *TLR7* and *TLR8* polymorphisms for HCV chronic infection and control groups.

	HCV	Control	p	OR
	N = 264	N = 243		(95%CI)
**TLR7 IVS2-151 (rs179009)**				
Male	N = 187	N = 146		
A	142 (75.9)^a^	125 (85.6)	**0.028** *	**1.89 (1.06–3.33)**
G	45 (24.1)	21 (14.4)		
Female	N = 77	N = 97		
AA	47 (61.0)	67 (69.1)	0.070	
AG	24 (31.2)	29 (29.9)	0.021#	8.55 (0.99–73.4)
GG	6 (7.8)	1 (1.0)		
A allele	118 (76.6)	163 (84.0)	0.149	1.45 (0.87–2.40)
G allele	36 (23.4)	31 (16.0)		
**TLR8 -129 (rs3764879)**				
Male	N = 187	N = 146		
C	33 (17.6)	10 (6.8)	**0.004** **	**2.91 (1.38–6.13)**
G	154 (82.4)	136 (93.2)		
Female	N = 77	N = 97		
CC	2 (2.6)	1 (1.0)	0.554	
CG	21 (27.3)	24 (24.7)	0.411#	
GG	54 (70.1)	72 (74.2)		
C allele	25 (16.2)	26 (15.5)	0.788	1.08 (0.61–1.88)
G allele	129 (83.8)	168 (84.5)		

a No (%) of subjects.

*P<0.05;

**P<0.001; compared with control group.

# Comparison of female subjects with homozygotes.

Based on haplotype analysis for homo- and hemizygous subjects, the frequency of wild TLR7 IVS2-151A/TLR8 -129G (AG) was significantly lower in chronic HCV infection patients than control subjects (65.3% versus 83.2%; p<0.001). Moreover, the haplotypes *TLR7 IVS2-151A/TLR8 -129C* (AC) and *TLR7 IVS2-151G/TLR8 -129G* (GG) increased the risk for chronic HCV infection compared to wild type *TLR7 IVS2-151A/TLR8 -129G* (AG), with OR = 2.67 (95% CI = 1.36 to 5.22; p = 0.003) and OR = 2.49 (95% CI = 1.43 to 4.34; p = 0.001), respectively (Table 2). Taken together, those with mutant variants of *TLR7* and *TLR8* had higher susceptibility to chronic HCV infection than those with wild type alleles.

**Table 2:** Haplotype frequencies of *TLR7* and *TLR8* genes for HCV chronic infection and control groups.

Haplotype	HCV	Control
	N = 225#	N = 220#
TLR7 IVS2-151A/TLR8 -129G (AG)	147 (65.3)*	183 (83.2)^a^
TLR7 IVS2-151A/TLR8 -129C (AC)	30 (13.3)*	14 (6.4)
TLR7 IVS2-151G/TLR8 -129G (GG)	44 (19.6)*	22 (10.0)
TLR7 IVS2-151G/TLR8 -129C (GC)	4 (1.8)*	1 (0.5)

# Heterozygous female individuals are excluded.

a No (%) of subjects.

*P<0.05; HCV group was compared with control group.

### TLR7 and TLR8 expression studies

**Figure 1:**
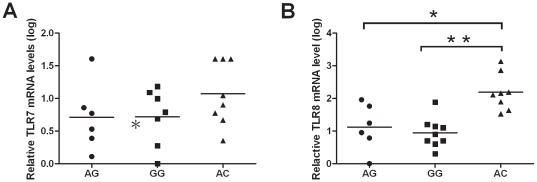
Quantitative TLR7 and TLR8 mRNA expression of individuals with different genotype by RT-PCR. The total RNA was extracted from buffy coat of male volunteers. The relative expression of TLR7 (A) and TLR8 (B) mRNA was reverse transcribed and obtained by using the 2^(−ΔΔCT)^ method which was normalized with an endogenous control, glyceraldehydes-3 phosphate dehydrogenase (GAPDH). Each dot represents an individual, (•) represent individuals with TLR7 IVS2-151A/TLR8 -129G genotype, (▪) represent individuals with TLR7 IVS2-151G/TLR8 -129G genotype and (▴) represent those with TLR7 IVS2-151A/TLR8 -129C genotype. The horizontal bars represent mean value. *: p-value<0.05. **: p-value<0.001.

Figure 1 shows the results of quantitative determinations for *TLR7* and *TLR*8 mRNA expressions in healthy male volunteers with different *TLR7* and *TLR8* haplotypes. Individuals with the *TLR7 IVS2-151A/TLR8 -129C* (AC) haplotype had higher TLR7 and *TLR8* mRNA expressions. *TLR8* mRNA expression for those with the AC wild type was significantly higher than for those with the AG and GG haplotypes (p = 0.008; 0.001; respectively).

**Figure 2:**
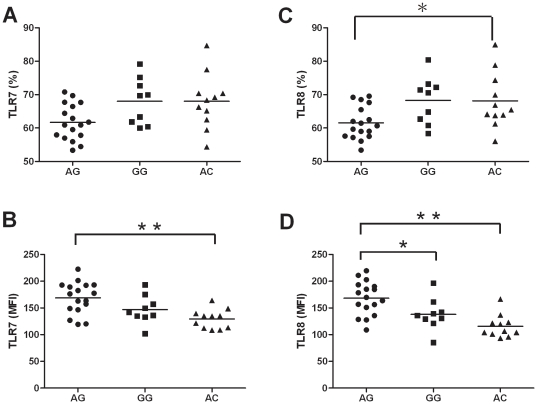
Intracellular TLR7 and TLR8 protein expression in monocytes of health male subjects with different genotypes of TLR7 and TLR8. The TLR7 and TLR8 protein expressions in monocytes of different genotypes from health male subjects were assessed by FACS. By double staining strategy with anit-CD14-PE conjugated antibody and monoclonal FITC conjugated TLR7 or TLR8 antibodies. (A) TLR7 expression represented as percentage of CD14-positive cells; (B) TLR7 expression represented as MFI; (C) TLR8 expression represented as percentage of CD14-positive cells; (D) TLR8 expression represented as MFI. Each dot represents an individual, (•) represent individuals with TLR7 IVS2-151A/TLR8 -129G genotype, (▪) represent individuals with TLR7 IVS2-151G/TLR8 -129G genotype and (▴) represent those with TLR7 IVS2-151A/TLR8 -129C genotype. The horizontal bars represent mean value. *: p-value<0.05. **: p-value<0.001.

Figure 2 shows the results of FASC analysis for the intracellular TLR7 and TLR8 protein expressions in monocytes from 37 healthy male subjects with different *TLR7* and *TLR8* genotypes: AG haplotype, N = 17; GG haplotype, N = 9; AC haplotype, N = 11. Those with the AG haplotype had a significantly lower percentage of CD14+ cells that expressed TLR7 (61.7±5.4%) compared to those with the GG haplotype (68.0±6.9%; p = 0.089) and the AC haplotype (68.0±8.3%; p = 0.066; Figure 2A). Also, the percentage of CD14+ cells from subjects with the AG haplotype that expressed TLR8 (61.5±4.9%) was significantly lower compared to cells from those with the GG haplotype (68.3±7.1%; p = 0.060) and the AC haplotype (68.1±8.3%; p = 0.048; Figure 2C). In contrast, the mean fluorescence intensities of TLR7 and TLR8 expressions were significantly higher in cells from subjects with the AG haplotype compared to cells from those with the GG and AC haplotypes (Figure 2B & 2D).

### *Ex vivo* stimulation assays

**Figure 3:**
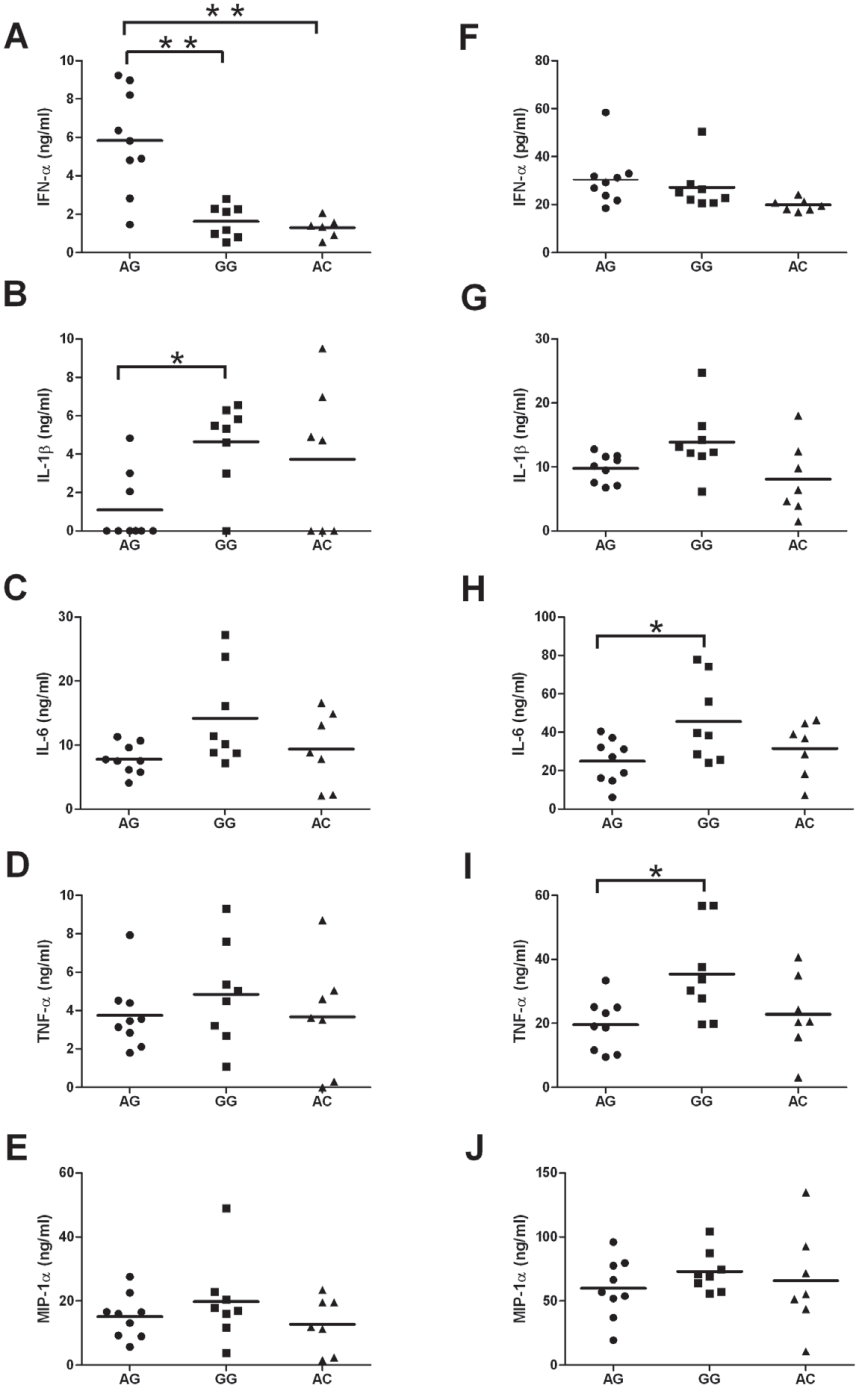
*Ex vivo* cytokines production upon TLR7 or TLR8 agonist stimulation during whole blood culture. Fresh whole blood specimens from male volunteers were stimulated with TLR7 agonist (3M-001) (A–E) or TLR8 agonist (3M-002) (F–J) for 12 hr. The concentrations of IFN-α, IL-1β, IL-6, TNF-α, and MIP-1α concentrations in the supernatant were measured by high sensitive IFN-α ELISA and Luminex100 system. Each dot represents an individual, (•) represent individuals with TLR7 IVS2-151A/TLR8 -129G genotype, (▪) represent individuals with TLR7 IVS2-151G/TLR8 -129G genotype and (▴) represent those with TLR7 IVS2-151A/TLR8 -129C genotype. The horizontal bars represent mean value.

To examine whether the *TLR7* and *TLR8* SNPs affected cytokine induction, we stimulated whole blood samples from healthy donors using TLR7 and TLR8 specific agonists. Whole blood was obtained from healthy male volunteers with different haplotypes and exposed *ex vivo* to a TLR7-specific agonist (3M-001) or a TLR8-specific agonist (3M-002) for 12 hours, after which the supernatants collected for cytokines assays. In response to 3M-001, whole blood from those with the wild type (AG) haplotype had higher IFN-α production as compared to the GG and AC haplotypes (p = 0.003 and 0.004, respectively; Figure 3A). However, lower IL-1ß production from those with AG haplotyp compared to the GG haplotype (p = 0.015; Figure 3B). Those with the AG haplotype exhibited decreased pro-inflammatory responses compared to the other haplotypes, as significant differences were observed for IL-6 and TNF-α after 3M-002 stimulation (p = 0.050 and 0.037, respectively; Figure 3G and 3I).

## Discussion

Although an antiviral immune response is staged in almost all patients infected with HCV, only some of these patients achieve a spontaneous resolution and the factors that determine the quality of these immune responses remain mostly unknown [16]. In addition to viral factors, host factors, including age, gender and genetic factors, influence the spontaneous outcome after HCV-infection, the progression to chronic disease and the response to therapy [17]. Thus, a role for TLR7 and TLR8 in the immune response against HCV, a single-stranded RNA virus, is conceivable, as an antiviral effect of TLR7 stimulation has been demonstrated in early clinical studies [13].

Our results showed that the *TLR7IVS2-151G* and *TLR8-129G* polymorphisms were statistically significantly more frequent in male subjects with chronic HCV infection as compared to controls. In addition, our results suggest that the *TLR7 (IVS2-151A>G)* and *TLR8 (-129G>C)* polymorphisms also altered these gene expressions when quantified by mRNA. However, *ex vivo* stimulation assays demonstrated that these *TLR7* and *TLR8* polymorphisms resulted in significantly different production of IFN-α, IL-1ß and IL-6 after stimulation with a TLR7 agonist, whereas only IL-1ß and TNF-α production were altered after stimulation with a TLR8 agonist (Figure 3). Taken together, the immune cells from those with the wild type AG haplotype produced more IFN-α and less amounts of pro-inflammatory cytokines upon stimulation. We propose the variations in the *TLR7* and *TLR8* genes may impair the immune responses to HCV due to less IFN-α production during stimulation.

The distributions of *TLR7 IVS2-151G>A* (rs179009) and *TLR8-129G>C* (rs3764879) allelic frequencies in other populations are shown in Table 3. In our Taiwanese study population, the genotype distributions for the *TLR7 IVS2-151G>A* and *TLR8-129G>C* polymorphisms were: 413 had the A allele (15.0%), 73 had the G allele (85.0%), and 46 had the C allele (9.5%), and 440 had the G allele (90.5%), respectively. Compared with other ethnic populations from the website of the international HapMap project, statistical analysis did not reveal a difference in allele frequencies for the *TLR7 IVS2-151G>A* polymorphism when comparing our population with Caucasians and Japanese, but there was a significant difference between our results and those from a Nigerian population (p = 0.009).

**Table 3:** Allele frequencies of TLR7 and TLR8 polymorphisms in different ethnic groups.

Gene SNP (dbSNP No)				
	Nigeria	Caucasian	Japanese	Taiwanese
***TLR7*** ** (X allele) IVS2-151 G>A (rs179009)**				
A allele	0.750*	0.817	0.807	0.850
G allele	0.250	0.183	0.193	0.150
***TLR8*** ** (X allele) -129G>C (rs3764879)**				
C allele	0.725**	0.775**	0.273**	0.095
G allele	0.275	0 .225	0.727	0.905

Allele frequencies of these ethic groups were retrieved from web of International HapMap project.

*P<0.05,

**P<0.001, comparing non-Taiwanese ethnic group with Taiwanese.

In our previous study, SNPs resulting in a Met/Val change at the start codon for *TLR8* and a (G/C) SNP at position −129 in the *TLR8* promoter region were shown to be in linkage [9]. As compared to other ethnic populations, Taiwanese exhibited an allele frequency for *TLR8* Met1Val and *TLR8* -129 G/C opposite to those of Caucasians and Nigerians (Table 3). The *TLR8* -129 G/C linkage with the *TLR8 A1G* polymorphism alters the start ATG of TLR8 into a GTG triplet. A methionine located at position 4 could be used as an alternate start codon, resulting in a truncated TLR8 (1038 aa vs. 1041 aa) with a shorter signal peptide. The functional effect of this mutation may result in a more rapid decay of *TLR8* mRNA or may affect protein function [18].

In overexpression assays, it has been demonstrated that a *TLR8 A1G* polymorphism resulted in impaired NF-κB activation in vitro. The mutated receptor variant was associated with modulating the cytokine secretion profiles and lipid mediator synthesis patterns in monocytes and neutrophils [18]. The functional TLR8 variant may have implications for assessing the risks for individual patients infected with HIV and other RNA viruses [18]. In this study we also demonstrated that those with the *TLR8-129G* associated *TLR8* variant had a higher susceptibility to HCV infection. This is consistent with previous studies [19], [20].

Undesired self-destructive immune responses result in a loss of a robust immune response against HCV replication and perturbations in virus eradication [21], [22]. An increasing body of evidence suggests a role for innate immunity in the control of HCV infection, although the precise mechanisms have not been defined. The potency of an innate immune response is genetically predetermined and can result in HCV clearance [22]. TLR7 and TLR8 are PRRs of interest in the setting of HCV-infection as they can bind ssRNAs and lead to the production of large amounts of the antiviral cytokine interferon-α by dendritic cells [23]. Therefore, we hypothesized that genetic variants of TLR7 and TLR8 might influence the immune response against HCV.

We selected TLR7- and TLR8-specific agonists for whole blood *ex vivo* stimulation. Whole blood samples from subjects with the wild type AG haplotype had higher IFN-α production as compared to the GG and AC haplotypes upon stimulation with 5 µM of 3M-001, but there was no induction of IFN-α after stimulation with the TLR8 agonist 3M-002 regardless of haplotype. The reason may be that plasmacytoid dendritic cells in peripheral blood, which express TLR7 but not TLR8, are the primary producers of IFN-α [23].

In contrast, upon stimulation with 5 µM of 3M-002, whole blood samples from those with the AG haplotype exhibited decreased pro-inflammatory responses reflected by IL-1β and TNF-α induction. High levels of these cytokines have been shown to be correlated with increased liver damage in HCV patients [16], [17], [24], [25]. Thus, male volunteers with the *TLR7 IVS2-151A/TLR8 -129G* haplotype had higher induced interferon production and lower amounts of inflammatory cytokines than those with the GG and AC haplotypes after *ex vivo* whole blood stimulation with TLR7 and TLR8 agonists.

The allele frequencies of both variations were gender-dependent. Because the *TLR7* and *TLR8* genes are located on the X-chromosome [4], gender-specific effects come as no surprise. The skewing of X-chromosomal activation in women is an age- and tissue-dependent process that may affect the analysis of the effects of X-chromosomal variations [15], [26]. Therefore, associations between chronic HCV chronic infection and *TLR7* and *TLR8* polymorphisms were not found among females in our study. Due to the accumulating data on genetic polymorphisms in HCV-infection [22], [23], [24], we suggest that patient's sex needs to be taken into account when future individual risk profiles for HCV infection are generated.

Our results suggest that *TLR7* and *TLR8* variants have functional relevance in the setting of HCV-infection by conferring susceptibility to infection. The *TLR7* and *TLR8* variants result in reduced IFN-α release. Accordingly, this could be responsible for a lower level of immune activation and explain the higher susceptibility to chronic HCV chronic infection among males with these mutations. Thus, our results support the concept that HCV infection depends on TLR7/8-mediated immune activation. It will be of interest to observe the results from further trials on the use of TLR agonists, such as isatoribine or CpG-DNA [13], [27], for the treatment of chronic HCV infection and how these are correlated with the genetic markers of the associated genes.

A different approach for investigating the roles of *TLR7* and *TLR8* SNPs in the resolution of HCV-infection would require a comparison of patients who are chronically infected with HCV with individuals that have spontaneously cleared infections. Unfortunately, patients with proven spontaneous recovery from HCV-infection were not included in this study. Therefore, we investigated the effects of these mutations in healthy volunteers. In humans, TLR7 is expressed by plasmacytoid DCs and B cells [28], [29], whereas TLR8 is abundant on monocytes and monocyte-derived DCs [30], [31]. Our results showed that subjects with the *TLR7 IVS2-151A/TLR8 -129G* haplotype had a lower percentage of monocytes that expressed TLR7 or TLR8, but these cells had higher intensity expressions compared to cells from those with other genotypes.

In conclusion, the results presented here demonstrate a protective effect of the *TLR7 IVS2-151A/TLR8 -129G* haplotype against HCV chronic infection. We showed that the presence of this mutation leads to lower IFN-α and higher pro-inflammatory cytokine production. The modulation of immune responses is not only compatible with the restriction of HCV infection, but leads us to propose that future studies should address the relevance of these polymorphisms during the clinical course of HCV infection. The results of this study may have implications for assessing individual patient's risk profiles, as well as for the use of TLR agonists in the prevention of or in therapy for HCV infection.

## Materials and Methods

### Study participants

We recruited a population of 264 chronic hepatitis C patients (including 187 men and 77 female adults) according to suggestions by Lu *et al* [32] ( mean age 52.9±15.7 years) and 243 control subjects (including 146 men and 97 female adults) who were anti-HCV negative ( mean age 48.3±19.8 years) for determinations of *TLR7* and *TLR8* gene variations. Patients with HBV or HIV infection were excluded. Chronic HCV infection was proven by detection of anti-HCV or HCV RNA in the patient's sera over a period of at least 6 months. Serum activity of aspartate aminotransferase (AST) and alanine aminotransferase (ALT) were recorded. The detail demographic characteristics of study subjects are summarized in Table S1. Informed consent was obtained from all individuals. This study was approved by the E-DA Hospital ethics committee.

### Genotyping of TLR7 and TLR8 variants

Genomic DNA was extracted from EDTA blood using spin columns with a QIAamp® Blood Mini Kit (Qiagen, Hilden, Germany). Genotyping for the *TLR7 (IVS2-151A>G)* and *TLR8 (-129 G>C)* polymorphisms were analyzed by polymerase chain reaction-restriction fragment length polymorphism (PCR-RFLP) as previously described [9].

### Determinations of TLR7 and TLR8 mRNA expressions by quantitative reverse transcription polymerase chain reaction (RT-PCR)

Healthy male volunteers (laboratory staff members and college students) were randomly selected from among healthy male subjects after *TLR7 IVS2-151* and *TLR8-129* genotyping. Total RNA was extracted from peripheral blood samples using a Qiagen RNA Blood isolation kit (Qiagen, Hilden, Germany) according to the manufacturer's instructions. Complementary DNA (cDNA) was synthesized by reverse transcription with oligo-dT primers. The mRNA expressions of *TLR7* and *TLR8* were analyzed by quantitative RT-PCR. Fluorescence real-time PCR analysis used an ABI PRISM 7700 Sequence Detection System (Applied Biosystems, Warrington, WA). The PCR amplification reaction used the conditions: 50°C for 2 min, 95°C for 15 min, and 40 cycles at 95°C for 15 sec followed by 58°C for 1 min. We used the comparative CT (ΔΔ*C*T) method, in which *C*T is the threshold cycle number that is the minimum required for sample detection. The arithmetic formula for the Δ*C*T method is the difference in CT for the *TLR7* or *TLR8* gene and a housekeeping gene, *GAPDH* (Δ*C*T = CT^TLR7^ or CT^TLR8^-CT^GADPH^). Then, the relative amounts of mRNA were normalized to the lowest *TLR7* or *TLR8* mRNA level given by 2^−ΔΔCT^ and given as the logarithm of 2^−ΔΔCT^.

### Intracellular TLR7 and TLR8 protein expression assay

The intracellular protein expressions of TLR7 and TLR8 in monocytes from healthy male volunteers with different haplotypes were analyzed by flow cytometric analysis. First, 250 µL of freshly collected EDTA-whole blood was stained with 5 µL of PE-conjugated anti-CD14 antibody (BD Biosciences, San Jose, CA) for 30 min at room temperature. Red blood cells were lysed by adding 2 mL of working 1× BD FACSTM Lysing Solution into each reaction tube for 10 min at room temperature, and then excess unbound antibody was washed away with phosphate buffered saline (PBS). For intracellular staining, the remaining leukocytes were fixed and permeabilised by adding 250 µL of Fixation and Permeabilization Solution (BD Biosciences, San Jose, CA) for 20 min at 4°C, after which cells were incubated with 2 µL of FITC-conjugated anti-TLR7 (IMGENEX, San Diego, CA) or anti-TLR8 (IMGENEX, San Diego, CA) diluted with 18 µL of 10% foetal bovine serum in PBS containing 0.2% saponin for 1 hr at 4°C. Cells were finally resuspended in PBS and analyzed on a FACSCalibur flow cytometer with WinMDI98 software (BD Biosciences, San Jose, CA).

### Cytokines released after ex vivo whole blood stimulation

Incubation of human whole blood in the presence of different stimuli was as described previously [33]. Briefly, fresh heparinised blood from healthy volunteers was diluted 2-fold with Hank's balanced salt solution (HBSS). The blood was incubated with synthetic specific agonists prepared by 3M Pharmaceuticals (St. Paul, MN) for TLR7 [34] (5 µM 3M-001, *N*-[4-(4-amino-2-ethyl-1H -imidazo[4,5-c]quinolin-1-yl)butyl]methanesulfonamide; formula, C17H23N5O2S; m.w., 361) and TLR8 [34] (5 µM 3M-002, 2-propylthiazolo[4,5-c]quinolin-4-amine; formula, C13H13N3S; m.w., 243) for 12 hours in the presence of 5% CO_2_ at 37°C. Then, the cells were pelleted by centrifugation (400× *g* for 2 min), and the cell-free supernatants were stored at −70°C prior to cytokine determinations. All experiments and measurements were carried out blindly with regard to the polymorphism status of the donors.

Cytokine amounts were quantified using Luminex technology, which utilizes microspheres as the solid support for conventional sandwich immunoassay to simultaneously measure multiple cytokines. The combinations of different types of beads allows for the simultaneous measurements of IFN-α, IL-1β, IL-6, TNF-α, and MIP-1α (Biosource International, Camarillo, CA). The cytokine kit assay used the manufacturer's protocol. Briefly, to each designated pre-wetted well with a filter bottom in a 96-well microplate format (Millipore, Billerica, MA), a bead suspension was added, and the beads were washed and blocked for 10 min with PBS/bovine serum albumin. To generate a standard curve, appropriate standards were prepared in diluents. Standards and samples (50 µl) were diluted 1∶1 with assay diluents and pipetted into duplicate wells. The plates were incubated for 1 h at room temperature on a microtitre shaker. After washing with PBS, a phycoerythrin (PE)-conjugated detection antibody cocktail was added to each well and the plates were incubated for an additional 45 min in the dark. Wells were washed twice, an assay buffer was added to each well, and samples were analyzed using the Luminex system (Bio-Rad Laboratories, Hercules, CA). The experimental data were analyzed using a five-parameter standard curve that was fit to the results for the provided standards.

### Statistical analysis

Genotype frequencies of *TLR7* and *TLR8* polymorphisms were compared between chronic HCV-infected patients and control subjects using chi-square tests. Quantitative results from three groups are given as means ± SD and assessed with nonparametric Kruskal-Wallis test. A p-value<0.05 was considered statistically significant. SPSS (version 14.01) was used for data management and statistical analyses and Graph Pad Prism 5 software was used to generate the figures.

## Supporting Information

Table S1 **Demographic characteristics of study subjects.**
(DOCX)
