# Supplementary material for: TLR7 and TLR8 Gene Variations and Susceptibility to Hepatitis C Virus Infection
Source: PLoS One. 2011 Oct 13;6(10):e26235. doi: 10.1371/journal.pone.0026235 (PMC3192790; doi:10.1371/journal.pone.0026235)
Supplement: Table S1 — Demographic characteristics of study subjects. (DOCX) [file pone.0026235.s001.docx]

**Table S1. Demographic characteristics of study subjects**

|  | **HCV**  **N = 264** | **Control**  **N= 243** |
| --- | --- | --- |
| Gender |  |  |
| Female | 77 (29.2)^a^ | 97 (39.9) |
| Male | 187 (70.8)* | 146 (60.1) |
| Age  Mean ± SD（years） | 52.9 ± 15.7* | 48.3 ± 19.8 |
| AST  Mean ± SD（U/L） | 74.3 ± 71.4** | 23.0 ± 10.3 |
| ALT  Mean ± SD（U/L） | 90.8± 111.5** | 35.1 ± 26.4 |

^a^ No (%) of subjects

* P < 0.05; ** P < 0.001; compared with control group
